# Analysis of postoperative pain after single versus multiple-visit root canal treatment in molars

**DOI:** 10.6026/973206300214849

**Published:** 2025-12-15

**Authors:** Priyatam Karade, Jasmine Marwaha, Shrutika Jadhav, Deepankar Dass, Mohd Ahmed Ali khan, Anisha Dandapat

**Affiliations:** 1 Department of conservative dentistry and endodontics, Bharati Vidyapeeth (Deemed To Be University) Dental College and Hospital, Sangli 416414, Maharashtra, India; 2 Department of Conservative dentistry and Endodontics, National Dental College and Hospital, Mohali-140507, India; 3 Department of Conservative dentistry and Endodontics, National Dental College and Hospital, Mohali-140507, India; 4 Department of Conservative Dentistry & Endodontics, Maharshi Markendeshwar College of Dental Sciences & Research, Mullana, Haryana, India; 5 Department of Conservative dentistry and endodontics, Rungta college of dental science and research, Chattisgarh, India; 6 Kalinga Institute of Dental Sciences, KIIT Deemed To Be University, Patia, Bhubaneswar, Odisha, India

**Keywords:** Endodontics, postoperative pain, root canal treatment, single-visit, multiple-visit, molars, visual analog scale

## Abstract

The number of visits to root canal treatment is still controversial especially on postoperative pain in molars. Therefore, it is of interest to compare pain outcome of single-visit and multiple-visit root canal treatments in 120 cases of molar root of a tooth. The intensity of pain was measured throughout 7 days with the help of the Visual Analog Scale, analgesic use and treatment success was also measured. Single-visit cases depicted a small difference in pain at 6 hours, but not after and the same level of success at 6 months. Single and multiple visit treatment offer similar postoperative comfort and superior clinical outcomes.

## Background:

Root canal treatment is one of the most widespread operations to be conducted in the field of modern dentistry with the main goal being the removal of infection and avoidance of reinfection of the root canal system [1]. The clinical treatment of endodontic therapy has significantly changed in recent decades and there is still controversy about the number of appointments that can be made to ensure successful endodontic practice [2]. Conventionally, the root canal treatment was done in several sessions, which provided the opportunity to use inter-appointment medication and reduce the level of bacteria slowly [3]. Nonetheless, the improvement of instrumentation methods, irrigation procedures and obturation sources has enabled completion of endodontic care in one session [4]. The potential benefits of the single-visit approach are that treatment time is reduced, there is a minimal chance of inter-appointment contamination, temporary restoration failures are eliminated and patient convenience will be increased [5]. On the contrary, advocates of multiple-visit treatment claim that the staged procedures can be better controlled in terms of infection, downplay the extrusion of debris and possibly it can result in lower incidence of postoperative complications [6]. Postoperative pain is one of such complications and still a major issue to both clinicians and patients, as it has an impact on the quality of life and may even influence treatment outcomes [7]. The cause of postoperative pain after root canal treatment is due to some intricate responses of mechanical instrumentation trauma, chemical irritability of irrigants and medicaments, microbial factors and personal patient factors [8]. The level of instances of postoperative pains has been documented to be between 3 and 58 percent based on numerous factors such as type of tooth, pulpal diagnosis and treatment protocol [9]. Their complicated anatomy, numerous canals and high occlusiveness pose special issues in the endodontic therapy as the molars might affect the postoperative pain reactions [10]. Some systematic reviews have tried to explain whether there is an effect of appointment number on the intensity of postoperative pain [11, 12]. The outcomes are however not consistent and some studies have indicated no significant effects [13, 14] whereas some have indicated slightly higher pain on single treatment visit [15]. This heterogeneity is due to methodological differences such as use of varying tools of pain assessment, follow up time and populations of patients [16]. New studies based on standard procedure and tested pain measurement tools have presented stronger evidence [17, 18]. However, in modern literature, there is still a lack of special examination of the topic of postoperative pain in molar teeth, which are relevant to a significant fraction of endodontic cases and have unique anatomic implications [19]. In addition, the majority of the available literature has been dedicated to the short-term measurement of pain and fewer have examined the patterns of pain resolution after 48 hours [20]. Since the importance of appointment number in treatment planning is clinical and there is still uncertainty about the effects of postoperative pain outcomes when it comes to molars, this prospective randomized controlled trial was set to be a comprehensive comparison of the relevance of pain intensity, prolongation and related clinical outcomes after single-visit versus multiple-visit root canal therapy in mandibular and maxillary molars. Our hypothesis was that single visit treatment would yield the same amount of postoperative pain as multiple visit treatment in the use of the standardized contemporary endodontic protocols. Therefore, it is of interest to analysis of postoperative pain after single-visit versus multiple- visit root canal treatment in molars.

## Materials and Methods:

The required sample size was determined using the G + Power software version 3.1 on which the pilot study indicated the mean VAS pain scores of 3.2 + 1.6 on a single visit and 2.6 + 1.4 on a multiple visit. With the assumption that = 0.05, = 0.80 and = 0.40, at least 50 patients in each group would be needed. To take into consideration the probability of 20% dropouts, 60 patients were recruited in each group (n=120).

## Participant Selection:

## Inclusion criteria:

Patients aged between 18-65 years old and need root canal therapy in mandibular or maxillary molars with vital pulp or asymptomatic irreversible pulpitis; diagnosis of cold test and electric pulp testing; teeth with closed apices and intact coronal structure with which rubber dam isolation is feasible; patients capable of comprehending VAS and giving informed consent; systemically healthy patients (ASA I or II).

## Exclusion criteria:

Teeth with symptomatic apical periodontitis or acute apical abscess; pulp necrosis with periapical radiolucency greater than 5mm diameter; teeth with open apices, perforations, or root resorption; previously treated endodontically; severe periodontal disease (probing depth more than 5mm); pregnant or lactating women; known hypersensitivity to local anesthetic or non-steroidal anti-inflammatory drugs; patients with systemic conditions which may affect pain perception.

## Randomization and allocation concealment:

Computer generated random numbers were used in sealed opaque envelopes prepared by an independent administrator to randomly assign the eligible patients to one of the single visit groups (SV) or multiple visit groups (MV). The process of allocation was done just prior to the initiation of treatment. Because of the specifics of the intervention, it was not possible to blind the operators, but the outcome assessors who analyzed the pain data were not aware of the allocation.

## Treatment protocol:

The three experienced endodontists (>10 years-experience) carried out all the procedures following standardized protocols. It was done under 2% lidocaine containing 1: 200,000 epinephrine (inferior alveolar nerve block of mandibular molars; infiltration of maxillary molars). High-speed diamond burs with lots of water coolant were used to prepare access cavity after rubber dam isolation. Electronic apex locator (Root ZX, J. Morita) was used to determine working length which was confirmed radiographically (established 0.5-1.0mm short of radiographic apex). The canal preparation was carried out with ProTaper Next rotary files (Dentsply Sirona) in a sequence, X1, X2, X3 and X4 according to canal anatomy. Irrigation procedure involved sodium hypochlorite 2.5% (3ml in between instruments) and end irrigation with 17% EDTA (2 minutes) followed with 2.5% sodium hypochlorite. The overall volume of irrigation was about 20ml/ Canal.

## Single-visit group:

After preparation and irrigation of canals, they were dried using paper points. Warm vertical compaction technique using AH Plus sealer (Dentsply DeTrey) and gutta-percha was used to obtain obturation. Composite resin (Filtek Z350 XT, 3M ESPE) was used to restore access cavity the same appointment.

## Multi-visit group:

Inter-appointment medication (UltraCal XS, Ultradent) placed was calcium hydroxide paste after canal preparation and irrigation. Temporary restoration placed (Cavit G, 3M ESPE) was used to close access cavity. The completion was done after 7 days. Calcium hydroxide was also extracted at the second appointment through irrigation with sodium hypochlorite 2.5% and hand files. Final restoration and obturation was done with the same material and techniques used in the single-visit group.

## Medication and postoperative instructions:

Patients were given equal instructions regarding the postoperative guidelines (not to chew on the treated tooth within the 24 hours, keep the mouth clean). Ibuprofen 400mg tablets were given to the patients and they were asked to take them when in pain only (rescue analgesic). Prescription of prophylactic antibiotics was not done.

## Outcome measures:

Primary outcome: Level of pain in 100mm Visual Analog Scale (VAS) where 0 = no pain and 100 = worst possible pain. Pain levels at 6, 12, 24, 48, 72 hours and 7 days after the treatment were noted in a standardized diary by the patients.

## Secondary results:

Analgesic use (number of ibuprofen tablets); at 7 days percussion sensitivity (present/absent); treatment success at 6 months (absence of clinical signs/symptoms and radiographic evidence of healing/stable periapical condition). They include the follow-up and the data collection. Each time point saw patients being contacted through the telephone to ensure that they complied with the recording of pain. At 7 days and 6 months, clinical examination was carried out. Success evaluation was done after 6 months when periapical radiographs were taken.

## Statistical analysis:

The SPSS version 26.0 (IBM Corp., Armonk, NY) was used to analyze the data. Shapiro Wilk test was used in the assessment of normal distribution. Independent t-test was used to compare demographic and baseline characteristics when the variables were continuous and chi-square test was used to compare demographic and baseline characteristics when the variables were categorical. Independent t-test was used to compare pain scores at various times of the day. To determine changes in pain, repeated measure ANOVA was used to compare the changes as time went by. The chi-square was used to compare analgesic consumption and percussion sensitivity. The level of statistical significance was defined as p <0.05. Mean ± standard deviation are used to present results in case of continuous variables and frequencies (percentages) in case of categorical variables.

## Results:

A total of 148 patients were assessed for eligibility, of which 120 met inclusion criteria and were randomized (60 per group). All patients completed the study with no dropouts. Table 1 (see PDF) presents demographic and baseline characteristics. Groups were comparable regarding age, gender, tooth location and preoperative diagnosis (p>0.05 for all comparisons). Table 2 (see PDF) presents mean VAS pain scores at different time points. At 6 hours postoperatively, the single-visit group demonstrated statistically significantly higher pain scores compared to the multiple-visit group (4.8 ± 1.9 vs 4.1 ± 1.7, p=0.041). However, this difference diminished rapidly, with no significant differences observed at subsequent time points: 12 hours (3.9 ± 1.6 vs 3.7 ± 1.5, p=0.482), 24 hours (2.8 ± 1.4 vs 2.6 ± 1.3, p=0.426), 48 hours (1.9 ± 1.1 vs 1.8 ± 1.0, p=0.594), 72 hours (1.2 ± 0.9 vs 1.1 ± 0.8, p=0.527) and 7 days (0.4 ± 0.6 vs 0.3 ± 0.5, p=0.338). Repeated measures ANOVA revealed significant reduction in pain over time in both groups (p<0.001), but no significant group x time interaction (p=0.186), indicating similar pain resolution patterns. Pain severity categories were further analyzed. At 6 hours, moderate to severe pain (VAS ≥4) was reported by 38 patients (63.3%) in the single-visit group versus 31 patients (51.7%) in the multiple-visit group (p=0.196). By 24 hours, this proportion decreased to 18 patients (30.0%) and 15 patients (25.0%) respectively (p=0.538). Table 3 (see PDF) presents secondary outcome measures. Analgesic consumption showed no statistically significant difference between groups. In the single-visit group, 47 patients (78.3%) consumed at least one dose of ibuprofen compared to 43 patients (71.7%) in the multiple-visit group (p=0.391). The mean number of ibuprofen tablets consumed was 2.1 ± 1.4 in the single-visit group and 1.9 ± 1.3 in the multiple-visit group (p=0.361). Percussion sensitivity at 7 days was minimal in both groups, present in 3 patients (5.0%) in the single-visit group and 2 patients (3.3%) in the multiple-visit group (p=0.648). Treatment success at 6-month follow-up was excellent in both groups. In the single-visit group, 58 patients (96.7%) achieved successful outcomes, while 59 patients (98.3%) in the multiple-visit group demonstrated success (p=0.559). The two failures in the single-visit group and one failure in the multiple-visit group required retreatment due to persistent symptoms and radiographic pathology. Exploratory subgroup analysis based on tooth location (maxillary vs mandibular) and pulpal diagnosis (vital vs asymptomatic irreversible pulpitis) revealed no significant interactions with treatment protocol regarding pain outcomes (p>0.05 for all comparisons). Gender-based analysis similarly showed no differential response to single-visit versus multiple-visit treatment (p=0.673).

## Discussion:

This was a prospective randomized controlled trial that involved comparison of the postoperative pain in molars undergoing single visit versus multiple visits root canal treatment with standardized contemporary protocols. The main conclusion was that single-visit treatment elicited pain levels that were much similar to multiple-visit treatment and there was only slightly high pain 6 hours after surgery that had no clinical significance. These two methods recorded good treatment results after 6 months. The temporary pain scores (6 hours) difference of 4.8 vs 4.1 (p=0.041) is a statistically significant but clinically insignificant difference of 0.7 points on the VAS scale. The result is in line with earlier studies that have found a marginally high level of acute postoperative pain after single-visit treatment [21, 22]. The mechanism which has been proposed is acute inflammatory response of the instruments and obturation done within a single visit which may lead to temporary exacerbation of periradicular tissue irritation [23]. The intense convergence of the levels of pain at 12 hours of time, however, indicates that this inflammatory reaction is self-regulated and well-bearable. Our findings support a number of recent systematic reviews that have established that appointment number is not found to have significant effects on postoperative pain [24, 25, 26- 27]. These results dispute the conventional beliefs that staged treatment can control inflammation. The pain history curves in the two groups showed increasing down even to almost disappearing at 7 days, which is in line with the normal postoperative healing curves [28]. This time pattern is the result of natural inflammatory cascade after endodontic instrumentation with initial acute inflammation giving place to resolution as periradicular tissues recovery [29]. The lack of notable group x time interaction in repeated measures analysis is a confirmation that there is identical healing kinetics irrespective of the number of appointments. The similarity of the two approaches is also backed through the analysis of the patterns of analgesic consumption. Most of the patients in the two categories needed rescue analgesia (78.3% vs 71.7%) and the average number of tablets taken was about 2 within the follow-up period. This little analgesic need implies that pain can be well managed under both of the protocols [30]. The consumption rates of analgesics have been reported to be inconsistent between 40 and 85 percent depending on the type of tooth and the diagnosis [31, 32]. Our results are also expected and illustrate no high analgesic load with single-visit treatment. The favorable outcomes of the treatment observed in the two groups (96.7% and 98.3%) are comparable to the modern-day success rates of primary endodontic therapy [33, 34]. These results dispel the historical anxieties that the single-visit treatment would dilute the success of long-term results because of the lack of disinfection [35]. Nickel-titanium instrumentation, better irrigation procedures and better obturation methods have allowed efforts in reducing bacteria and sealing the canal in 3D and one appointment, respectively [36]. Warm vertical compaction was used in this study to ensure maximum obturation quality, which is a determinant of success of treatment [37]. There are a number of reasons that could explain why single visit treatment was no better than multiple visit treatment in our study. First, the cases were all of vital or asymptomatic pulps without acute periapical pathology, which is a good situation to complete without any follow-up [38]. Second, the antimicrobial efficacy was guaranteed through the standardized irrigation with a sufficient volume of sodium hypochlorite (around 20ml per canal) [39]. Third, highly trained operators working in the best environment controlled the technical variables that could affect the results [40]. These findings have clinical implications. Single-visit treatment has been characterized by tremendous benefits such as less treatment time, doing away with inter-appointment complications (temporary restoration loss, bacterial recontamination), better patient convenience and possibly reduced overall cost [41, 42]. Considering similar pain and success results shown in this study, the single-visit treatment is a possible and effective method of treating the molars with vital or asymptomatic pulps. Such practice can be especially useful in the modern practice when the time spent on patients and their treatment efficiency becomes a matter of concern [43]. Nevertheless, there are a number of limitations which should be mentioned. First, the research population was limited to teeth with necrotic pulps and symptomatic apical periodontitis as they could react to the single-visit treatment differently [44]. Second, the work was carried out by the experienced endodontists and the results might be different with inexperienced operators [45]. Third, pain measurement was based on patient-reported results, which presented the possibility of recall bias even with structured diaries utilization [46]. Fourth, the six months follow-up, though sufficient to assess early success, may not be effective to assess late failures [47]. Sustained success equivalence requires further larger-scale follow-up studies. Future studies ought to examine the outcome of single-visit versus multiple-visit of teeth with necrotic pulps and periapical pathology, examine cost-effectiveness in terms of both healthcare system and patients, examine outcomes in general practice with the different levels of operator experience and examine biomarkers of inflammation which may predict the presence of postoperative pain [48, 49]. Also, patient preferences and satisfaction measures are significant outcome dimensions that should be evaluated systematically [50].

## Conclusion:

Single visit root canal therapy gives equivalent postoperative pain and success results to multiple visit therapy when administered using standardized modern techniques. The short-term nature of the early postoperative pain is clinically insignificant and disappears shortly. Both methods are effective and the choice of treatment can be made by considering the complexity of the case, expertise of the operator and the preference of the patient.
